# Heterotrophic inorganic carbon fixation varies with temperature and resource availability in marine and soil bacterial isolates

**DOI:** 10.1128/aem.00628-26

**Published:** 2026-09-28

**Authors:** Emma K. Brown, Kadin Pulliam, Katelyn Weeks, Carolynn M. Harris, William D. Leavitt, Jaclyn K. Saunders, Elizabeth Trembath-Reichert

**Affiliations:** 1 School of Earth and Space Exploration, Arizona State University 7864, Tempe, Arizona, USA; 2 Department of Geology & Geophysics, University of Utah 402023, Salt Lake City, Utah, USA; 3 Department of Marine Sciences, University of Georgia 1355, Athens, Georgia, USA; University of Minnesota Twin Cities, St. Paul, Minnesota, USA

**Keywords:** anaplerotic carboxylases, microbial carbon cycling, bacterial metabolism, nutrient limitation, stable isotope probing, heterotrophic inorganic carbon fixation

## Abstract

Inorganic carbon use in cellular metabolism is ubiquitous in all living organisms. The assimilation of inorganic carbon into biomass via heterotrophic inorganic carbon fixation (HICF) is estimated to contribute significantly to global carbon fixation, yet the controls on this process in strictly heterotrophic microorganisms remain poorly understood. To identify potential drivers of HICF, we cultivated well-characterized heterotrophic Gammaproteobacteria (*Pseudomonas putida* and *Pseudoalteromonas carrageenovora*) at two temperatures (optimal vs colder) and resource treatments (high vs low organic carbon), all of which were amended with NaH^13^CO_3_. We performed bulk and single-cell stable carbon isotope probing (SIP) analysis to detect and quantify HICF in each condition, and assessed other measures of metabolic state, including C/N ratio, natural abundance nitrogen isotope ratios, and cell size. We observed HICF in all treatments, with the percent of biomass originating from ^13^C-dissolved inorganic carbon (DIC) for *P. putida* (0.006% to 0.029%) and *P. carrageenovora* (0.18% to 1.1%) on the lower end of estimates for the contribution of anaplerotic fixation to heterotrophic biomass (1% to 8%). HICF was generally highest during exponential phase, at the optimal growth temperature, and under low carbon conditions, and these trends were also observed at the single-cell level. Overall, temperature and resource availability appear to influence HICF rates. These findings suggest that HICF may be more relevant under environmental conditions where DIC and nitrogen are not limiting, and the flux of digestible organic carbon is limited or intermittent. Environmental applications of ^13^C-DIC SIP in such systems may record heterotrophic contributions to community carbon fixation.

**IMPORTANCE:**

Heterotrophic microorganisms are abundant across ecosystems and play a central role in environmental carbon cycling by storing, transforming, and decomposing organic matter. However, heterotrophs can also incorporate inorganic carbon into biomass, but this is rarely considered in models of the carbon cycle. Our experiments on heterotrophic bacteria from soil (*Pseudomonas putida*) and marine environments (*Pseudoalteromonas carrageenovora*) show that inorganic carbon uptake is impacted by both resource availability and temperature and may be undetectable without stable isotope probing. By integrating single-cell techniques, our study measures variation in inorganic carbon uptake among individual cells across growth phases and conditions. Our study highlights heterotrophic inorganic carbon fixation as a variable source of ^13^C-dissolved inorganic carbon incorporation into microbial biomass, underscoring the importance of considering this process in the design and interpretation of environmental stable isotope probing studies.

## INTRODUCTION

Heterotrophs are defined by their need to assimilate organic carbon to survive. However, heterotrophs also utilize inorganic carbon via a range of carboxylation reactions. For example, inorganic carbon can be used to generate intermediates of the TCA cycle (i.e., anaplerosis) and to facilitate the synthesis of fatty acids, nucleotides, amino acids, and vitamins (1). Wood and Werkman (2) first described the utilization of inorganic carbon by a heterotroph (2), and subsequent laboratory studies suggest that 1% to 8% of biomass in heterotroph cells is derived from dissolved inorganic carbon (DIC) via anaplerosis (3–10). Assimilation of DIC into biomass attributed to heterotrophic prokaryotes has been observed in soil (11–15), lake (16, 17), marine (18–24), and deep subsurface environments (25–27). Overall, heterotrophic inorganic carbon fixation (HICF) is estimated to fix 0.34 to 12.3 Pg of DIC per year, which is on the same order as the amount of carbon annually produced by photoautotrophs (28).

**Fig 1:**
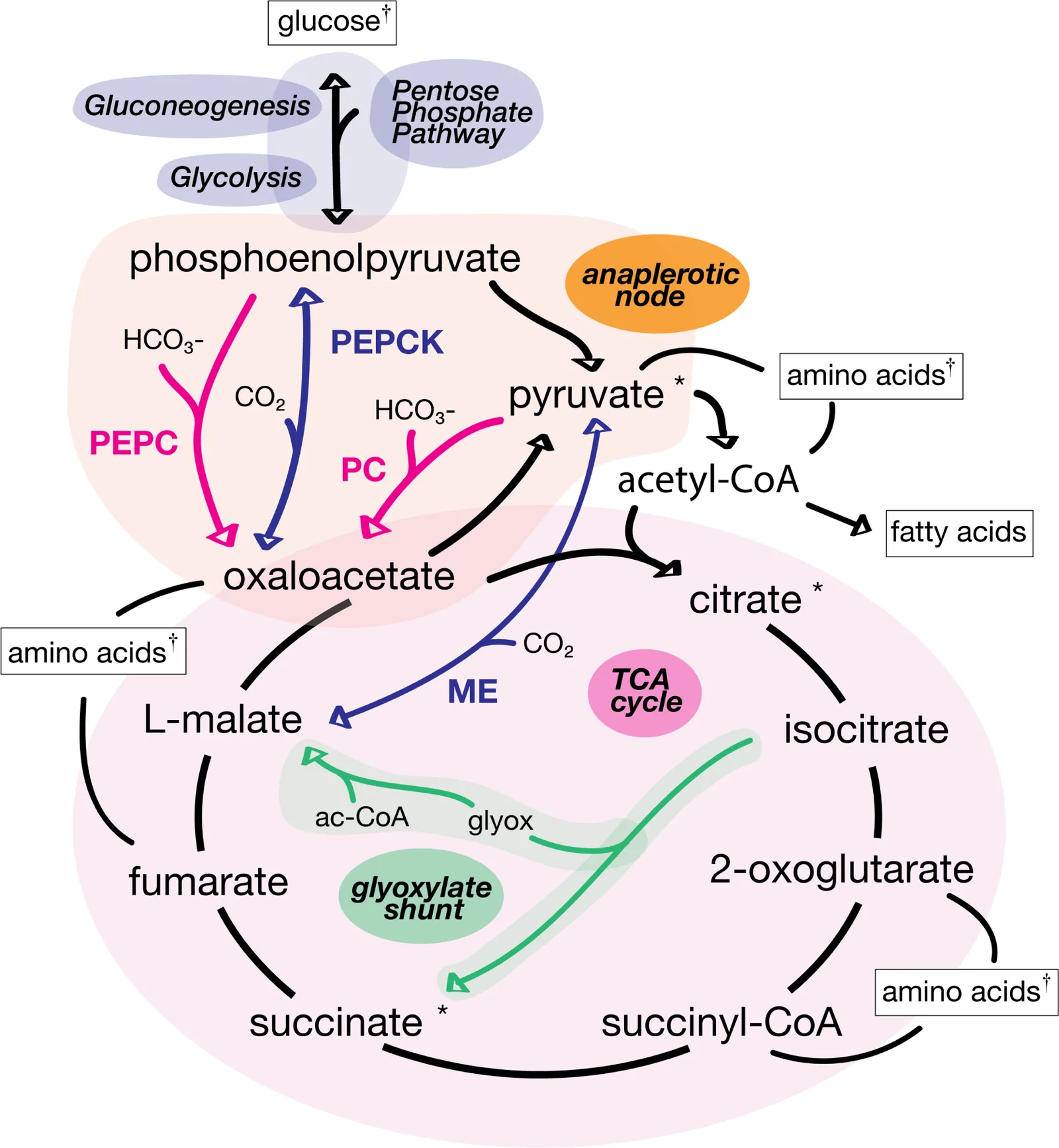
Overview of carbon flux through central carbon metabolic pathways. The anaplerotic node, known as a switching point between anabolism and catabolism, hosts key carboxylating enzymes, pyruvate carboxylase (PC) and phosphoenolpyruvate carboxylase (PEPC), in addition to the malic enzyme (ME) and phosphoenolpyruvate carboxykinase (PEPCK). ^*^Carbon substrates supplied in *P. carrageenovora* growth medium. ^†^*P. putida* growth medium contained glucose and tryptone, a source of amino acids. Amino acids can enter the TCA cycle at different points through various mechanisms.

Microbial HICF has been linked to increased expressions of anaplerotic carboxylases during seawater (20) and soil incubations (12). Anaplerotic carboxylases are essential for maintaining a functioning TCA cycle in heterotrophic microbes (29). Depending on the cell’s metabolic needs, carbon flux can be directed both in and out of the TCA cycle at the anaplerotic node via the carboxylation or decarboxylation of oxaloacetate, allowing the replenishment of TCA cycle intermediates or the generation of sugars through gluconeogenesis (Fig. 1). The direction of carbon flow through the central metabolism was recently observed to influence the magnitude of HICF in *Bacillus subtilis* (10). When grown on glucose as a sole carbon source, as opposed to malate (an intermediate of the TCA cycle), *B. subtilis* generated a higher percentage of biomass from DIC (10).

While several controlled laboratory investigations have sought to determine environmental factors that trigger or limit HICF, works to date have yielded conflicting results. Observations of DIC assimilation by *P. putida* and *E. coli* suggest that HICF is substrate-stimulated, and little to no HICF occurs in cells that are starving or metabolically inactive (7, 30). In contrast, *B. subtilis* supplied with NaH^13^CO_3_ during the stationary phase assimilated enough ^13^C-DIC to supplement 2% of its biomass (10), suggesting HICF can occur even when cell density is not increasing during stationary phase. Conflicting results are also reported for marine ecosystems. Organic carbon amendments to seawater have been shown to stimulate anaplerotic reactions by putative heterotrophs (20, 23), yet anaplerotic DIC fixation was proposed to be important for the survival of marine bacteria under organic carbon-deplete conditions (18, 22, 31). Additionally, changes in temperature, which affect growth rate and other cellular properties, may influence HICF. For example, DIC assimilation was observed in shallow crustal fluid incubations by putative heterotrophs, with more occurring at 20°C than at 4°C (25). It remains unclear how growth phase and environmental parameters, including resource availability and temperature, control HICF across diverse heterotrophic bacteria. This uncertainty is especially relevant for dark carbon fixation and ^13^C-DIC SIP studies, where it is difficult to deconvolve autotrophic, mixotrophic, and heterotrophic contributions to net rates of carbon fixation.

Despite these uncertainties, HICF is thought to be regulated by a cell’s anabolic demands. Inorganic carbon assimilation has even been investigated as a mechanism to track heterotrophic metabolic activity and growth in the environment (7, 32). Various degrees of coupling between anabolic and catabolic fluxes have been observed under different resource limitations in chemostat cultures of *B. subtilis,* where nitrogen limitation led to a higher flux through the pyruvate carboxylase than carbon limitation (33). A larger increase in anaplerotic carboxylase activity has also been observed in response to nitrogen limitation as opposed to carbon limitation in experiments with other aerobic heterotrophic bacteria (34). Though cellular C and N budgets are known to change in response to resource availability, how HICF fits into cellular responses is not well constrained. For example, the carbon-to-nitrogen (C/N) ratio in bacterial biomass has been shown to vary in response to resource stoichiometry (35–40) and growth rate (40–42). Specifically, bacteria cultured under nitrogen limitation have much higher C/N ratios than those cultured under carbon limitation (39, 41, 43). These differences are reduced at higher growth rates (40, 42). Nitrogen limitation has also been linked to larger cell size in heterotrophic bacteria, due to the accumulation of organic carbon stored as polysaccharides or polyhydroxyalkanoates (44–46). In turn, a decrease in cell size has been observed in heterotrophic bacteria during carbon limitation (38, 47, 48), thereby allowing more efficient nutrient transport and energy use by increasing the surface-to-volume ratio of the cell. While environmental resource imbalances can impose various morphological and C/N budget changes in the cell, whether and how HICF is affected by these conditions remain poorly constrained.

To investigate how resource limitation and temperature modulate HICF, we conducted axenic batch experiments amended with ^13^C-labeled NaHCO_3_ to enable HICF detection in a 2 × 2 factorial design (cool vs optimal temperature; high vs low organic carbon). We hypothesized that the amount of biomass generated from ^13^C-DIC would vary depending on resource condition, temperature, and growth phase. We tested the responses in two heterotrophic bacterial isolates from different ecosystems: *Pseudomonas putida* strain ATCC 17453, an aerobic soil microbe, and *Pseudoalteromonas carrageenovora* DSM 6820, an aerobic marine microbe. These strains are well-characterized obligate heterotrophs from the same taxonomic class (Gammaproteobacteria) that contain the phosphoenolpyruvate carboxylase (PEPC). Heterotrophic Gammaproteobacteria are consistently found to engage in DIC assimilation in marine and soil environments (12, 18–20). We performed bulk and single-cell carbon isotope analyses to explore mean DIC assimilation and population heterogeneity. In addition, we collected C/N ratio, nitrogen isotope, and cell size measurement data to strengthen the interpretation of our results and the connection to environmental data. Our study contributes to the growing understanding that all heterotrophs assimilate inorganic carbon to some extent and provides additional constraints on HICF environmental controls.

## RESULTS AND DISCUSSION

### *P. carrageenovora* growth and elemental composition

**Fig 2:**
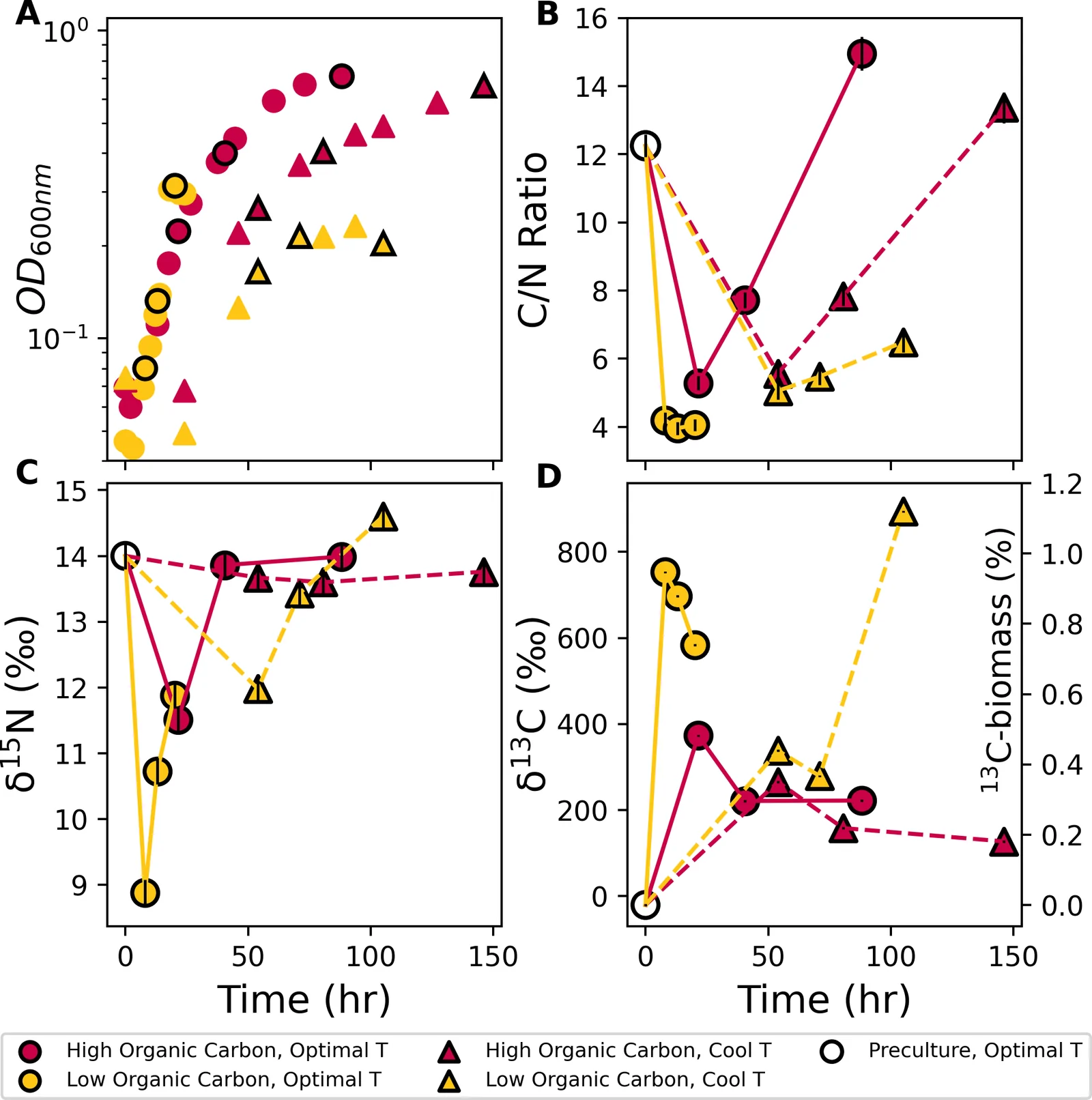
Batch experiments with *P. carrageenovora* grown in either the high OC condition (red) or low OC condition (yellow) at optimal temperatures (circles) or colder temperatures (triangles) amended with 2 mmolal ^13^C-labeled NaHCO_3_. Error bars represent the analytical precision calculated from technical replicates. (**A**) Averaged optical density measurements of culture replicates. The symbols outlined in black represent when cultures were harvested for EA-IRMS analysis. (**B**) C/N ratios of biomass. (**C**) δ^15^N (vs air) values of biomass. (**D**) δ^13^C (vs VPDB) values of biomass with the percent of biomass originating from ^13^C-DIC on the secondary *y*-axis (Eq. 2; Eq. 3).

Based on the framework that substrate C/N ratios can induce certain resource limitations (35, 49), high organic carbon (OC) cultures were expected to enter stationary phase due to nitrogen or phosphorus limitation, whereas low OC cultures were expected to be carbon-limited. *P. carrageenovora* was grown in medium with a substrate ratio of carbon, nitrogen, and phosphorus (C:N:P) of 3,113:24:1 (high OC condition) or in medium with a C:N:P of 311:24:1 (low OC condition). The high OC cultures had a higher yield (OD_max_: 0.71, 0.66) than the low OC cultures (OD_max_: 0.31, 0.23) at the optimal (24 ℃) and cool (14 ℃) temperatures, respectively (Fig. 2A). *P. carrageenovora* grew faster at the optimal temperature compared to the lower temperature, as expected (Table S1; Table S3). For high OC cultures, biomass C/N ratios more than doubled from exponential to stationary phase, reaching values of 13.4 and 15 at the optimal and cool temperature, respectively (Fig. 2B). Average cell size increased in parallel, which likely reflects the uptake and storage of excess OC into the cell in response to nitrogen or phosphorus limitation (Fig. S1; Table S4). High C/N ratios of bacteria limited by nitrogen or phosphorus can be explained by a decrease of protein content in the cell (50) and an increase of carbon for storage purposes (51), which has been observed in nature (52). In contrast, low OC *P. carrageenovora* cultures either experienced no discernible change in C/N ratio (4.2 to 4.1) throughout the growth curve (optimal T) or a slight increase from five at exponential to 6.5 at late stationary phase (cool T). Additionally, cells from the low OC condition did not increase in size and became more circular in stationary phase compared with cells from the high OC condition (Fig. S1; Table S4). Carbon limitation can induce such responses in cell morphology (53). The large increase in C/N ratios and cell size of *P. carrageenovora* in the high OC condition, in combination with the high substrate C/N for the media, suggests that the cells became increasingly nitrogen- or phosphorus-limited as they entered stationary phase. In contrast, *P. carrageenovora* in the low OC condition entered stationary phase due to carbon limitation.

### Isotopic evidence for nitrogen limitation in *P. carrageenovora* cultures grown with excess organic carbon

Nitrogen isotope data support nitrogen as the limiting resource under high OC conditions, rather than phosphorus. All *P. carrageenovora* samples harvested in the mid-exponential phase are depleted in δ^15^N_biomass_ relative to the preculture (harvested in late-exponential), consistent with the kinetic isotope effects during nitrate assimilation (54–56) (Fig. 2C). The δ^15^N_biomass_ values were less depleted in the cool T, high OC condition, likely because the 54-h sample was harvested after nitrogen became critically limiting. Weight percent nitrogen (Wt%N) from the cool T, high OC samples continuously decreased into stationary phase, whereas a decrease in Wt%N was not observed in the high OC, optimal T condition until the 80.5 hr sample (Fig. S2). The decrease in sample Wt%N in these two conditions is observed in tandem with steady δ^15^N_biomass_ values that resemble the preculture (14‰) and generic sodium nitrate (δ^15^N_NaNO3_ = 16.5‰ [57]). Reaching a uniform δ^15^N_biomass_ value in the stationary phase is expected if all the NO_3_^−^ was fully assimilated into biomass, assuming a closed system model (58). Together, these observations indicate that nitrogen—rather than phosphorus—is the limiting nutrient in the high OC condition for *P. carrageenovora*.

In contrast, δ^15^N_biomass_ values of low OC *P. carrageenovora* increased, suggesting they did not fully deplete nitrate in the media. Biomass at the cool temperature had the highest δ^15^N value in the stationary phase, becoming more enriched in ^15^N than the preculture, while also decreasing in Wt%N from late-exponential to the stationary phase. Optical density measurements suggest this sample is more representative of the late-stationary phase. Cells in the stationary phase employ various stress responses, including scavenging or the synthesis of secondary metabolites (59). If, as Dijkstra et al. proposed, biomass is enriched in ^15^N when carbon is limited and nitrogen-bearing organic molecules are repurposed, this would explain the simultaneous enrichment of ^15^N and decrease in Wt%N in low OC cultures into the late stationary phase (60).

### *P. putida* growth and elemental composition

**Fig 3:**
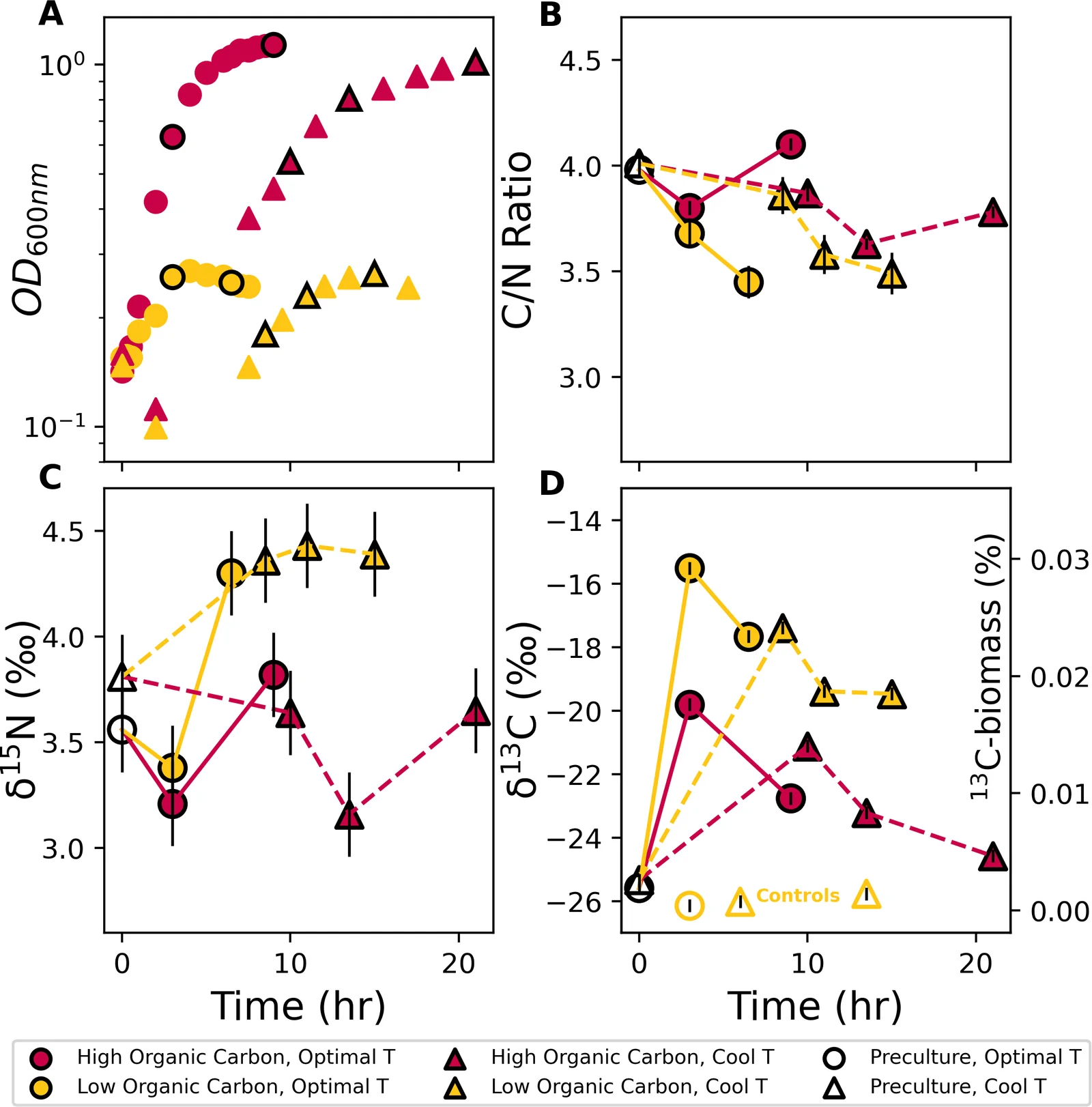
Batch experiments with *P. putida* grown in either the high OC condition (red) or low OC condition (yellow) at optimal temperatures (circles) or colder temperatures (triangles) amended with 15 μmolal ^13^C-labeled NaHCO_3_. Error bars represent the analytical precision calculated from technical replicates. (**A**) Averaged optical density measurements of culture replicates. The symbols outlined in black represent when cultures were harvested for EA-IRMS analysis. (**B**) C/N ratios of biomass. (**C**) δ^15^N (vs air) values of biomass. (**D**) δ^13^C (vs VPDB) values of biomass with the percent of biomass originating from ^13^C-DIC on the secondary *y*-axis (Eq. 2; Eq. 3).

High OC *P. putida* cultures reached a higher OD_max_ (1.13, 1.04) than the low OC cultures (OD_max_: 0.27, 0.24) at the optimal (30℃) and cool (15℃) temperatures, respectively (Fig. 3A), consistent with the *P. carrageenovora* experiments. *P. putida* cells from the high OC condition were also larger on average than cells from the low OC condition. However, cells from all growth conditions became more circular in the stationary phase, though this was more prominent for cells from the low OC condition (Fig. S1; Table S4). *P. putida* cells from a different strain have been shown to shift into a more coccoidal cell shape and decrease in size during carbon starvation, attributed to reductive cell division (48).

Patterns in *P. putida* carbon and nitrogen composition across growth conditions differ from those observed in *P. carrageenovora* experiments. Biomass C/N ratios were largely invariant among growth conditions (3.45 to 4.10; Fig. 3B) and resembled the C/N ratio of tryptone (~3.5 [61]). While tryptone was provided as the sole nitrogen source in *P. putida* media, it can also be a source of organic carbon. Therefore, tryptone concentrations were varied between OC conditions (see Methods) even though glucose was intended as a primary carbon source based on the original tryptone-glucose-yeast (TGY) medium used by the strain’s culture collection. However, the unexpectedly low biomass C/N ratios of high OC cultures and the preculture δ^13^C_biomass_ values did not support the use of glucose as a carbon source. For comparison, lipids from a different strain of *P. putida* grown with glucose had δ^13^C values (~ −12.5‰) close to that of glucose (−10.10‰) (62). Given the natural abundance δ^13^C values of our controls and precultures (−25‰ to −26‰) and peptone from casein digest (δ^13^C ~−23‰ to −27‰ [63, 64]), it appears tryptone was the preferred carbon source for *P. putida*. In fact, *P. putida* cultures failed to grow on glucose as the sole carbon source, and only began growing after tryptone amendment (Fig. S3). High OC biomass Wt%C and Wt%N values were 44% and 31% higher on average, respectively, than low OC biomass (Fig. S2). However, since cultures relatively higher in carbon were also higher in nitrogen, and vice versa, the C/N ratios of *P. putida* biomass varied <1 across growth conditions. C/N ratios did trend in opposite directions into stationary phase depending on the OC condition, alluding that substrate concentration, in addition to C/N, impacts cellular carbon and nitrogen budgeting.

### Isotopic evidence for resource partitioning in *P. putida*

Different trends were observed in the δ^15^N_biomass_ values of *P. putida* between high and low OC conditions, despite the two growth media having equal substrate C/N (Fig. 3C). Since *P. putida* cultures likely used tryptone (a mixture of peptides/various amino acids) as both a carbon and a nitrogen source, these cultures were more likely to exhibit deamination effects. The use of amino acids as a carbon source at low substrate C/N ratios occurs via deamination, resulting in the export of NH_3_ from the cell (65). Deamination can preferentially remove ^14^N-amino groups, subsequently enriching δ^15^N_biomass_ upon NH_3_ export (66, 67). Considering that all *P. putida* cultures were stoichiometrically limited by carbon, the mineralization of nitrogen-bearing organic material could explain the enrichment of ^15^N into the stationary phase. In fact, the subtle switch between nitrogen assimilation as a dominant mechanism versus dissimilation can be observed in the δ^15^N_biomass_ values of *P. putida* in the cool T, high OC condition, where δ^15^N_biomass_ was initially depleted during exponential phase, before becoming enriched during stationary phase (Fig. 3C). In contrast, δ^15^N_biomass_ values from the cool T, low OC condition remained constantly enriched compared to the preculture throughout the growth curve. The isotopic fractionation associated with N assimilation of organic compounds can be balanced by N dissimilation when they occur at similar rates (60). Higher δ^15^N_biomass_ values for *P. putida* biomass in the low OC condition could be explained if a larger fraction of the tryptone was mineralized in this condition than in the high OC condition, suggesting that cellular carbon and nitrogen budgeting depend on more than just substrate C/N ratios.

Overall, carbon appears to be the limiting resource in both high- and low-OC conditions for *P. putida* experiments. Little variation in the biomass C/N ratio across growth conditions, the low tryptone C/N ratio, and trends in cell morphology support this claim. In turn, unique trends in C/N and δ^15^N_biomass_ values between growth conditions are attributed to differences in substrate availability.

### Heterotrophic ^13^C-NaHCO_3_ uptake observed in both Gammaproteobacteria strains

All *P. carrageenovora* and *P. putida* cultures across all treatments were enriched in ^13^C when cultivated with an inorganic ^13^C-carbon substrate (Fig. 2D; Fig. 3D). This observation is consistent with the prediction that heterotrophic microorganisms can incorporate inorganic carbon into biomass. However, the amount of biomass originating from ^13^C-DIC for *P. putida* (0.006% to 0.029%) and *P. carrageenovora* (0.18% to 1.1%) varied depending on the growth condition and was on the lower end of previous estimates of HICF contributions due to anaplerosis (1% to 8% [28]). Given the potential for dilution of ^13^C in the DIC pool throughout the experiment due to respiration, our reported values are likely underestimated. A sensitivity analysis approximating the magnitude of this effect is described in the Supplemental Text and discussed further in Section 2.8.

**Fig 4:**
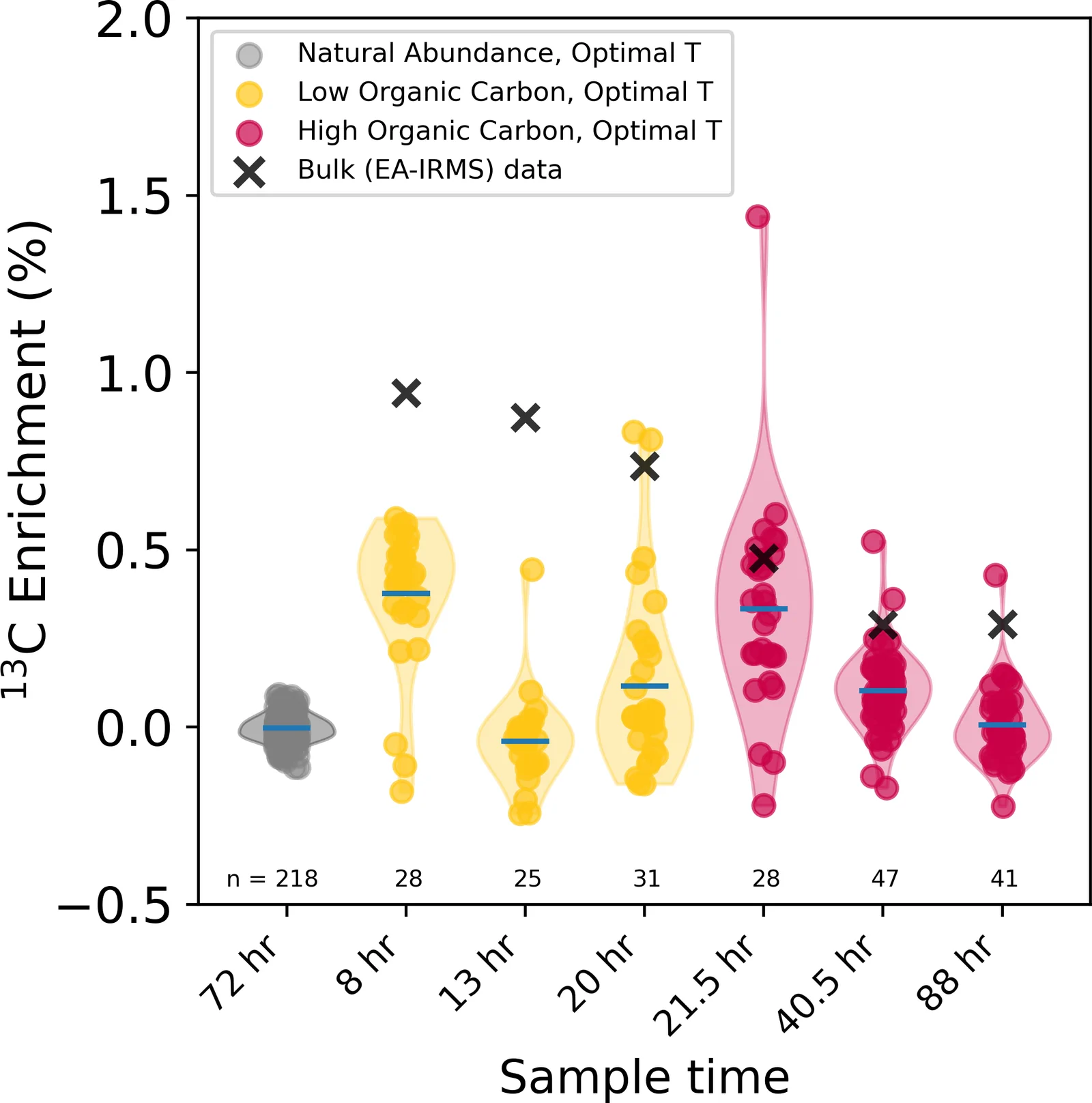
Single-cell ^13^C/^12^C analysis of *P. carrageenovora* samples from batch experiments amended with 2 mmolal ^13^C-labeled NaHCO_3_ in either organic carbon-rich media (red circles) or organic carbon-poor media (yellow circles) at the optimal growth temperature (24°C). Cells in unlabeled media were harvested in the late exponential phase for natural abundance single-cell ^13^C/^12^C ratios (gray). Percent ^13^C enrichment, plotted on the *y*-axis, is calculated for each analyzed cell using Eq. 4. Blue bars represent the average %^13^C enrichment in each sample, and the number of cells analyzed (*n*) is denoted at the bottom of each violin. Black x’s represent the %^13^C enrichment from EA-IRMS of the corresponding bulk sample.

Single-cell carbon isotope measurements were performed on select *P. carrageenovora* samples to evaluate heterogeneity in HICF that would be masked by bulk isotope analysis (Fig. 4). The average ^13^C fractional abundance of the unlabeled *P. carrageenovora* cells was 0.02165 with a variance of 0.0052 (*n* = 218) and was used in the calculation for single-cell %^13^C enrichment (Eq. 4). We observed cells that were more ^13^C-enriched than natural abundance cells in all the experimental samples analyzed. Calculated single-cell DIC incorporation rates from exponential phase samples overlap with those observed in the crustal fluid biosphere (0.042 to 3 fmol C per cell per day [25, 68]) (Fig. S4).

Average single-cell %^13^C enrichment values were highest in the exponential phase samples (0.33% high OC, 0.38% low OC; optimal T)—an observation reflected in bulk δ^13^C_VPDB_ values from EA-IRMS. Similarly, both methods found cells in the low OC conditions to be more ^13^C-enriched than high OC cells at this time point. However, trends from later time points differed between bulk and single-cell analyses. For example, a continuous decrease in ^13^C enrichment was observed in bulk isotope data for the low OC condition, but single-cell analysis revealed more ^13^C-enriched cells in the stationary phase compared to the mid-exponential sample. This stationary phase sample had a higher population variance (0.2; *n* = 31) in single-cell ^13^C enrichment than samples collected earlier in the growth curve for the low OC condition. In contrast, population variance decreased continuously throughout the growth curve in high OC samples. Heterogeneity in *E. coli* cells has been observed to increase after starvation (69). If similar dynamics were occurring in *P. carrageenovora* cultures, this could explain the increase in variance in ^13^C enrichment among single cells cultivated under the low OC condition compared with the high OC condition. Overall, single-cell ^13^C/^12^C analysis revealed heterogeneity in pure culture samples that is not captured in bulk isotope analysis.

The average ^13^C-DIC enrichment per sample from single-cell analysis was lower than the corresponding sample’s ^13^C enrichment calculated from EA-IRMS data (Fig. 4). In fact, we observed cells that were more depleted in ^13^C than the range of natural-abundance single-cell ^13^C/^12^C ratios in all experimental samples. These observations can be partially explained by dilution of ^13^C from the use of unlabeled chemicals during sample preparation (e.g., PFA), which has been shown to dilute ^13^C in a NanoSIMS analysis of bacterial cells (70–72). Further discrepancy may be explained by the probable heterogeneous nature of both PFA and label distribution within a given cell (e.g., *where* the labeled molecules are in the cell) that cannot be fully captured in small cells (70). The offset between single-cell and bulk measurements may be amplified in our study due to the small fraction of ^13^C-labeled biomass in the cells compared to enrichment values from studies using ^13^C-labeled organic carbon substrates for heterotrophic bacteria.

### Organic carbon availability influences HICF

**Fig 5:**
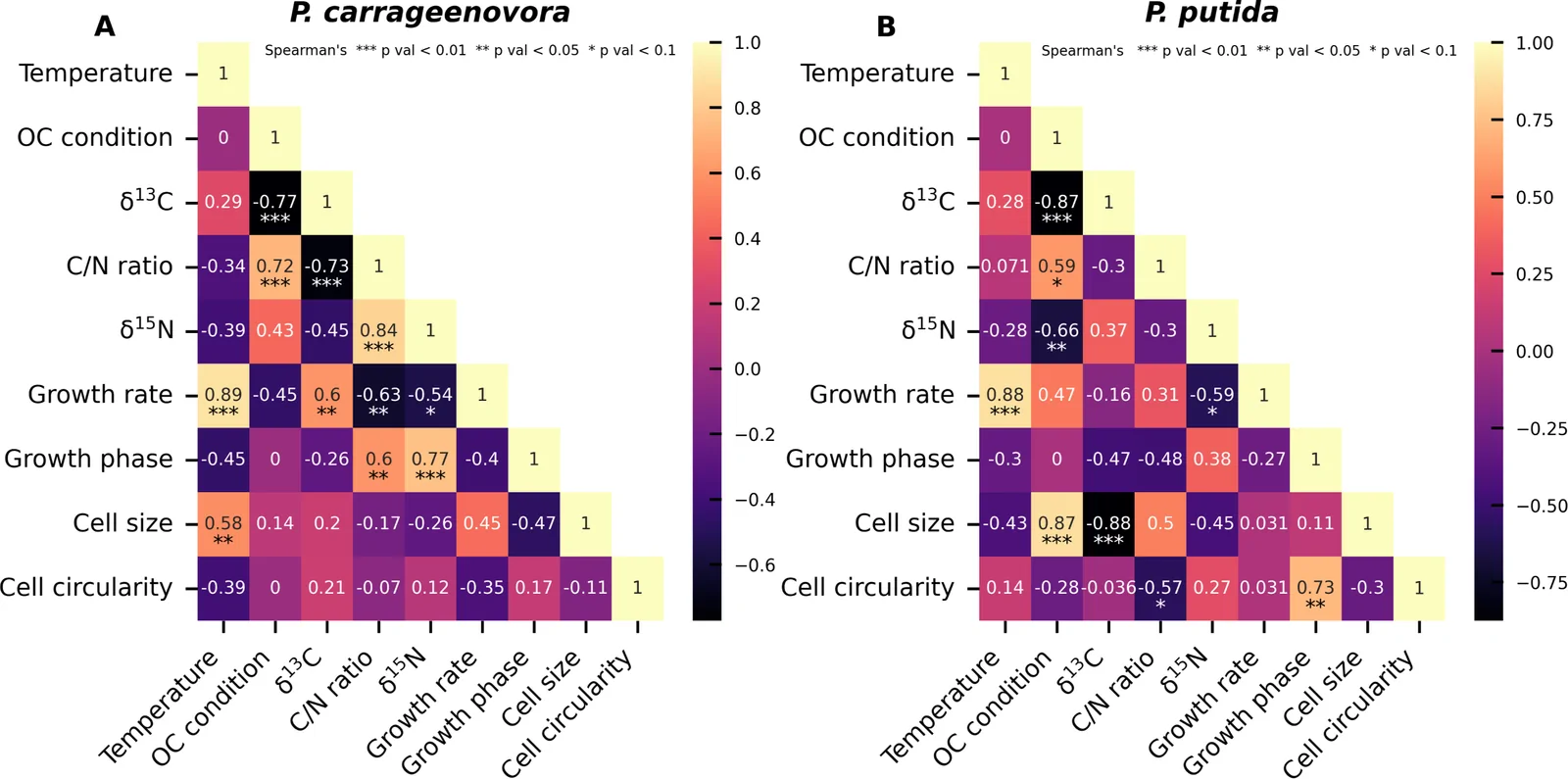
Correlation matrix generated using Spearman’s rank correlation coefficients for (**A**) *P. carrageenovora* and (**B**) *P. putida* experimental data. Asterisks represent statistically significant correlations.

Different levels of heterotrophic inorganic carbon fixation (HICF) by the two bacterial strains were observed. While both bacterial strains performed HICF, the magnitude of ^13^C-DIC incorporation varied depending on the concentration of organic carbon in the media, temperature, and growth phase. To compare the relative influence of experimentally manipulated growth variables (e.g., temperature and OC condition) on HICF, Spearman’s rank correlation coefficients were calculated (Fig. 5). Of all variables tested, the concentration of organic carbon has the strongest correlation with δ^13^C values for both *P. carrageenovora* (rho = −0.77, *P*-value = 0.003) and *P. putida* (rho = −0.87, *P*-value = 0.001).

Our results indicate that carbon limitation, as determined by substrate C/N ratio, is a driver of HICF in the experiments and that its impact is concentration-dependent. In general, more ^13^C-DIC assimilation occurred when either strain was provided with less organic carbon (Fig. 2D; Fig. 3D). This is inconsistent with predictions based on the higher expression of anaplerotic carboxylases in nitrogen-limited conditions as opposed to carbon in other aerobic heterotrophic bacteria (33, 34). In turn, our results agree with the notion that anaplerotic DIC fixation is important for heterotrophic prokaryotes in organic carbon-deplete marine systems (18, 22, 31). Contributions to HICF through mechanisms other than anaplerosis are also a plausible consideration. While both high and low OC conditions for *P. putida* experiments were considered carbon-limited, more ^13^C-DIC assimilation was observed in the low OC condition. This pattern was recently observed in dark soil incubations, where greater DIC fixation attributed to anaplerosis was observed in soils with low C/N ratios and low carbon and nitrogen concentrations (13). Similarly, increased ^13^C-DIC assimilation in organic carbon-limiting conditions was observed in mixotrophic growth of an aerobic bacterium (5) and in heterotrophic growth of fungi (73). In the former study, no difference in ^13^C-DIC assimilation was observed when bacteria were cultivated heterotrophically with 7.28 or 4 mM glucose, although the chemostat dilution rates differed, complicating the comparison (5).

### Temperature may impact HICF

Temperature showed a moderate association with culture δ^13^C values to approximately the same degree for both strains (rho ~+0.28, *P*-values > 0.3) (Fig. 5). We observed more ^13^C-DIC assimilation when *P. carrageenovora* and *P. putida* were cultivated at their optimum temperature (24°C and 30°C, respectively) as opposed to when cultivated at a colder temperature (Fig. 2D & Fig. 3D). This result is consistent with predictions based on higher activity of anaplerotic enzymes at optimal growth temperature compared with colder temperatures (74, 75) and an increase in DIC assimilation by putative heterotrophs with increasing temperature in shallow crustal fluid incubations (25). Though laboratory conditions differ from natural systems, these observations together suggest temperature may impact HICF.

### ^13^C-DIC uptake as a function of growth phase

By sampling throughout the growth curve, our study aimed to investigate potential relationships between growth phase and HICF. Growth phase was found to trend negatively with culture δ^13^C values, though this was more apparent for *P. putida* (rho = −0.47; *P*-value = 0.17) than *P. carrageenovora* (rho = −0.26, *P*-value = 0.41) (Fig. 5). In *P. putida* experiments, we observed a decrease in ^13^C enrichment in microbial biomass from exponential to stationary phase. In fact, in the cool T, high OC condition, the δ^13^C value of *P. putida* biomass at stationary phase (−24.58‰) almost returned to the preculture value (−25.34‰), compared to its value at exponential phase (−21.11‰). This pattern is not observed in the cool T, low OC condition, where the δ^13^C value remains constant during the transition from exponential to stationary phase (~ −19‰).

*P. carrageenovora* biomass harvested from exponential phase was also more enriched in ^13^C than biomass from late-exponential or stationary phase, but this trend did not hold for the low OC condition at the cool temperature. The release of unlabeled DIC from respiration would progressively dilute ^13^C in the DIC pool over the course of the growth curve. To explore the potential magnitude of this dilution and its impact on HICF estimations, we performed a sensitivity analysis across a range of carbon use efficiency (CUE) values (76) (supplemental text). Depending on the amount of respiration, HICF could be underestimated by approximately 1.01-2.16x in low OC conditions and 1.06-3.87x in high OC conditions for *P. carrageenovora* experiments (Fig. S10). HICF values were more sensitive to respiration in *P. putida* experiments due to the low starting concentration of ^13^C-DIC in the medium (Fig. S14).

While isotope dilution obfuscates the interpretation of HICF levels, a decrease in metabolic activity as the bacteria entered stationary phase likely led to less DIC assimilation. This observation is consistent with the findings that HICF is correlated with active growth (7, 30). However, the increase in ^13^C enrichment of *P. carrageenovora* biomass in the late stationary phase in the cool T, low OC condition does not follow this paradigm. The optical density decreased from the previous time point, while the C/N ratio increased and the δ^15^N_biomass_ became enriched, surpassing the preculture value. These trends may be due to the scavenging or repurposing of nitrogen-bearing biomolecules for energy or other maintenance operations during carbon starvation in the stationary phase. High DIC uptake in the stationary phase was observed in marine incubations where 70 to 80% of the cells were heterotrophic Gammaproteobacteria taxa (18). Furthermore, enzyme activity of PEP carboxylase in a marine bacterium was found to increase by a factor of 2.6 in stationary phase compared to exponential phase (77). The authors speculated this was in response to ongoing or anticipated carbon limitation (77). The δ^13^C value of *P. carrageenovora* biomass at late stationary phase (893‰) is ~2.6 times higher than during exponential phase (339‰) in ^13^C-DIC labeling experiments. If carbon limitation in stationary phase resulted in a higher activity of the PEP carboxylase, as opposed to nitrogen limitation, that could explain the increased uptake of ^13^C-DIC by *P. carrageenovora* when cultivated in the low OC condition at the cool T, but not in the high OC condition at either temperature. The last sample from the optimal T, low OC condition was taken just as the culture reached maximum optical density and is therefore not representative of late stationary phase. Further investigations of HICF across growth phases, including studies specifically targeting metabolic activity, are warranted.

### Biochemical and enzymatic considerations for HICF

Several factors relevant to this study design have been shown to influence HICF, including the type of organic carbon used in the media and the concentration of DIC. For example, an isotope-labeling study with 1 g/L NaH^13^CO_3_ showed that *B. subtilis* supplements 5% to 6% of its biomass with DIC when grown on lactate or glucose, respectively, but supplements only 3% of its biomass when grown on malate, which is an intermediate of the TCA cycle (10). *P. carrageenovora* medium contained two intermediates of the TCA cycle—citrate and succinate—which directly replenish the cycle, reducing the amount of anaplerotic carbon required and, consequently, lowering ^13^C-DIC assimilation. Therefore, HICF occurring at a reduced magnitude when two intermediates of the TCA cycle are provided in the *P. carrageenovora* medium agrees with findings in Spona-Friedl (10).

While *P. putida* medium lacked TCA cycle intermediates, it did contain tryptone, a rich source of amino acids and peptides. Amino acids like L-glutamate and L-aspartate, both of which are abundant in tryptone, can be transformed into intermediates of the TCA cycle, such as 2-oxoglutarate and fumarate. Growth on amino acids as a sole carbon source could result in flux into the TCA cycle to produce PEP, feeding the GNG/PP pathways with less drawdown of intermediates for the synthesis of biomolecules like amino acids (Fig. 1). For example, when *E. coli* was grown on amino acids, genes encoding for enzymes involved in GNG (including PEPCK) and those encoding amino acid catabolism enzymes were significantly up-regulated compared to when glucose was provided (78). However, when *P. putida* and *E. coli* were cultivated on a reduced-strength tryptone soy broth medium containing amino acids in the presence of 2.5–7.5 mM ^13^C-NaHCO_3_, ^13^C-enriched lipids were observed from both *P. putida* (up to ~120‰) and *E. coli* (up to ~950‰) (7). The concentration of NaH^13^CO_3_ in the medium was over two orders of magnitude higher than that used in our study, and the specific *P. putida strain* was not reported for direct comparison with our strain (7).

Further considerations for the levels of HICF observed in this study include the enzymatic capabilities of the two isolates. In addition to anaplerotic carboxylation, assimilatory and biosynthetic carboxylation provide additional mechanisms for DIC incorporation into biomass (1). In a survey of available genome annotations, we found that *P. carrageenovora* genome contained a greater number of carboxylating enzymes than *P. putida* (Table S7). For example, *P. carrageenovora’s* genome contains the 3-methylcrotonyl-CoA carboxylase and its growth medium provides the essential metals (e.g., Mg^+2^) and cofactors (e.g., biotin) for this carboxylase. This additional mechanism for DIC assimilation could increase HICF in *P. carrageenovora*. However, biotin supplementation could also lead to reduced HICF levels by decreasing the activity of dethiobiotin synthetase in *de novo* biotin synthesis given the exogenous source. Our survey did not identify assimilatory carboxylases in *P. putida* ATCC 17453. Future studies combining stable isotope probing with gene expression assays are needed to decipher the contribution of carboxylases to HICF across diverse isolates.

The very low percentage of ^13^C-DIC-enriched biomass observed in *P. putida* experiments may be a factor of the DIC concentration within the growth medium. The concentration of NaH^13^CO_3_ in the medium was ~15 μmolal to minimize altering the standard growth medium (see Materials and Methods). Low levels of intercellular inorganic carbon have been shown to impair anaplerotic flux in aerobic heterotrophic growth of *Corynebacterium glutamicum* (79). Furthermore, assimilation of DIC by heterotrophic bacteria is positively influenced by CO_2_ concentration (9). A possible explanation for these observations concerns the regulatory properties and cofactors of the PEPC enzyme, among other carboxylating enzymes. Higher bicarbonate concentrations have been shown to increase PEPC activity in assays (80). Though plant extracts were used for the study, the effect of bicarbonate was proposed to be independent of phosphorylation (80). In addition to low concentrations of one of the substrates (HCO_3_^−^) for the carboxylation reaction, a bivalent metal cofactor (Mg^+2^ or Mn^+2^), necessary for PEPC (81) and other carboxylating enzymes (1), was also likely present in low concentrations, given the *P. putida* medium ingredients. We conclude that the relatively lower ^13^C-DIC assimilation observed in *P. putida* batch experiments can be explained by a combination of the minimal PEPC substrate (DIC) and cofactor concentration in the growth medium, few other carboxylating enzymes, and the use of amino acids as a carbon and nitrogen source.

### Conclusion

We conducted batch experiments with a soil bacterium (*P. putida*) and a marine bacterium (*P. carrageenovora*) that varied in resource condition (high vs low organic carbon) and growth temperature (cool vs optimal) to identify potential drivers of HICF. We found that the assimilation of ^13^C-DIC into biomass occurred under all growth conditions, including low DIC concentrations, excess organic carbon conditions, and both conditions combined. In general, more HICF was detected under carbon-limitation, during exponential phase, and at the optimal growth temperature. We observed several markers of carbon limitation in stationary phase samples (low C/N ratio, small cell size, relatively high δ^15^N) from cultures that were stoichiometrically limited by organic carbon and fixed more DIC, compared to markers of nitrogen limitation, such as high C/N ratio. Our results for *P. carrageenovora* suggest HICF is reactivated in stationary phase when the cells are limited by carbon, but not nitrogen, and this response appears to be strain-dependent. Furthermore, our study demonstrates the use of NanoSIMS to identify ^13^C-DIC assimilation at the single-cell level in axenic heterotrophic cultures. We found that general trends in the bulk biomass δ^13^C were generally reflected at the single-cell level, despite the low ^13^C enrichment and number of cells analyzed per sample. Our laboratory single-cell DIC incorporation rates coincide with putative HICF rates in the crustal fluid biosphere (25, 68). These observations posit heterotrophic contributions to community DIC uptake rates, highlighting the importance of considering HICF when interpreting ^13^C-DIC SIP and dark carbon fixation studies. Overall, our findings suggest that HICF is a responsive process with organic carbon availability and temperature emerging as modulators, and we provide constraints that can guide the design of future laboratory and environmental studies on HICF.

## MATERIALS AND METHODS

### Strain and culturing information

Batch experiments were performed with two heterotrophic bacteria: *Pseudomonas putida* (ATCC 17453, JCM 6157), a soil microorganism purchased from the United States Department of Agriculture’s ARS Culture Collection, and *Pseudoalteromonas carrageenovora* (DSM No. 6820), a marine microorganism purchased from the Leibniz Institute DSMZ—German Collection of Microorganisms and Cell Cultures. *P. putida* precultures were grown on a modified TGY medium containing (per liter): 1 g of K_2_HPO_4_, 5 g of tryptone, 22.5 g of glucose, and 150 μL of a 0.1 molal NaHCO_3_ stock solution. *P. carrageenovora* precultures were grown on a modified L1 medium containing (per liter): 19.45 g of NaCl, 5.9 g of MgCl_2_, 3.25 g of Na_2_SO_4_, 2.25 g of CaCl_2_× 2H_2_O, 0.55 g of KCl, 80 mg of KBr, 34 mg of SrCl_2_, 4 mg of Na-silicate, 2.4 mg of NaF, 0.5 mL of the f/2 vitamin stock solution, 2 mL of 3.15 g/L FeCl_3_ stock solution, 1 mL of 75 g/L NaNO,_3_ and 5 g/L NaH_2_PO_4_× H_2_O stock solution, 1 mL of a trace element mix (82), 30 mL of a mixed carbon solution (50 g/L Na-citrate, 50 g/L Na-pyruvate, 50 g/L Na-succinate), and 19 mL of a 0.1 molal NaHCO_3_ solution. All solutions and media were prepared using sterilized MilliQ water and autoclaved or filter-sterilized with a 0.22-μm syringe filter.

### ^13^C-labeled NaHCO_3_ experiments

For ^13^C-labeled experiments, the appropriate volume of a 0.1 molal 99% atom ^13^C-NaHCO_3_ (Cambridge Isotope Laboratories) solution was added to the respective media to match the concentration of bicarbonate in the unlabeled media (15 μmolal for *P. putida*; two mmolal for *P. carrageenovora*) for a final enrichment of 50 atom% and 99 atom% ^13^C-DIC, respectively. Since DIC is not explicitly added to *P. putida* media, we added the estimated concentration dissolved from the atmosphere, which doubled the total DIC concentration, resulting in a 50 atom% label. Each strain was grown in a 2 × 2 factorial design with high vs low organic carbon concentrations and optimal vs cool temperatures, yielding four growth conditions (Table 1). Selected substrate concentrations were determined to ensure that the low substrate condition (10% of the high substrate concentration) significantly restricted growth while still allowing adequate biomass accumulation for subsequent analyses. Precultures and ^13^C-labeled experimental cultures were all incubated shaking at 120 rpm in ThermoScientific Solaris 4000 R incubators. Triplicate precultures were combined at the late exponential phase, homogenized, and 5 mL was used to inoculate 45 mL of ^13^C-labeled media in 150 mL flasks capped with secured sterile foil. Growth was tracked by measuring the optical density of approximately 100 μL of each culture at 600 nm with a spectrophotometer (ThermoScientific NanoDrop 2000). Cultures were harvested in triplicate for sample analyses at three different time points throughout the growth curve, except for *P. putida* at 30°C. This experiment was performed first, and samples were collected in duplicate for this condition and only at two time points. We decided to collect at three time points and in triplicate for subsequent experiments to maximize biomass yields.

**TABLE 1:** Summary of growth conditions for *P. carrageenovora* and *P. putida* batch experiments amended with ^13^C-NaHCO_3_^*a*^

*P. carrageenovora*		*P. putida*	
High OC	Optimal T (24^o^C)	High OC	Optimal T (30^o^C)
Low OC	Low T (14^o^C)	Low OC	Low T (15^o^C)
High OC	Low T (14^o^C)	High OC	Low T (15^o^C)
Low OC	Optimal T (24^o^C)	Low OC	Optimal T (30^o^C)

^*a*^ Cultures were grown in a 2 × 2 factorial design, resulting in four growth conditions that varied in temperature (T) and concentration of organic carbon (OC).

Aliquots of *P. putida* and *P. carrageenovora* precultures were harvested to determine the natural abundance of ^13^C in the microbial biomass. 25 mL of the remaining preculture was inoculated into the respective ^13^C-labeled media, shaken in the incubator for 10 to 15 min, and then pelleted to account for any unincorporated label not removed by washing (preculture wash test; Table S5). Before the cultures were harvested, 400 μL of each culture was pipetted into 100 μL of 20% paraformaldehyde (PFA) in 2-mL Eppendorf tubes and left on the benchtop for an hour to fix. Cryovials were then placed in a 5°C fridge for further analysis (see Supplemental Methods). Harvested cultures were pelleted in a centrifuge (Eppendorf 5804 R) for 10 min at 4,000 rpm at the respective incubation temperature. Pellets were then washed by resuspension in 10 mL of 1× phosphate buffer solution (PBS) and spun for another 10 min at 4,000 rpm. After a secondary wash step, pellets were resuspended in 1 mL of 1× PBS, transferred to sterile 15-mL Falcon tubes, and immediately frozen at −80°C. Frozen pellets were freeze-dried for 24 h at 0.02 mTorr and −40°C using a Labconco freeze dryer.

### Elemental and bulk stable isotope analyses

Dried pellets were pooled with their respective replicates and then analyzed for ^13^C and ^15^N with a Thermo Finnigan thermochemical elemental analyzer coupled to a Thermo Fisher Scientific MAT253 isotope ratio mass spectrometer at Arizona State University. All samples and standards were packed into Costech tin capsules and weighed on a Sartorius CP2P microbalance. NIST SRM1573a Tomato Leaves were used to calibrate for carbon and nitrogen concentration. Carbon and nitrogen isotope calibration curves were generated from glycine standards, in addition to USGS41a and USGS40. The analytical precision was determined to be 0.1‰ for δ^13^C_VPDB_ and 0.2‰ for δ^15^N_Air_ by calculating the relative standard deviation of glycine mid standards. To account for signal saturation for more ^13^C-enriched samples, in-house ^13^C-enriched NaHCO_3_ standards (100, 500, and 1000‰) were added to the carbon isotope calibration. A *P. carrageenovora* sample with high ^13^C enrichment was divided into a triplicate to gauge the analytical precision of EA-IRMS for highly enriched samples. The standard error of the mean was calculated to be 1.44‰, which is applied to all samples that fall outside of the constrained ^13^C calibration curve (above 36.55‰). Carbon-to-nitrogen (C/N) ratios were calculated by dividing the weight percent of carbon by the weight percent of nitrogen, then multiplying by the ratio of the C and N atomic masses (1.167). The standard error of the mean for weight percent carbon and nitrogen was calculated to be 0.12% and 0.05% for *P. putida* samples and 0.32% and 0.04% for *P. carrageenovora* samples, respectively, from technical replicates (Table S5).

All ^13^C/^12^C and ^15^N/^14^N ratios are reported in delta notation (δ) in permil (‰) units relative to the international standard (R_standard_), Vienna PeeDee Belemnite (VPDB) or Air, respectively (Eq. 1). The percent of biomass originating from labeled ^13^C DIC was estimated using Eq. 2 and Eq. 3 below, where the moles of carbon in the biomass (n_c_total_) were multiplied by the ratio of the differences of fractional abundances of the sample (F_sample_) and ^13^C-labeled DIC (F_label_) to the organic substrate in the media (F_substrate_). To compare bulk EA-IRMS data to single-cell NanoSIMS data, the percent enrichment of ^13^C in samples was determined using relative isotopic fractional abundances of samples compared with the respective unlabeled preculture (F_NA_) and the isotope ratio of the dissolved inorganic carbon in the respective media (F_label_) using Eq. 4 below.


(1) $$\;\delta^{13}C\text{ or }\delta^{15}N=\left(\frac{R_{sample}-R_{standard}}{R_{standard}}\right)\times 1000,\;\text{where }R=\frac{{}^{13}C}{{}^{12}C}\text{ or }\frac{{}^{15}N}{{}^{14}N}$$

(2) $$n_{c\_inorg}=n_{c\_total}\times \left(\frac{F_{sample}-F_{substrate}}{F_{label}-F_{substrate}}\right)\text{ where }F=\frac{R}{R+1}$$

(3) $${\%}^{13}C\;labeled\;biomass=\left(\frac{n_{c\_inorg}}{n_{c\_total}}\right)\times 100$$

(4) $${\%}^{13}C\;enrichment=\left(\frac{F_{sample}-F_{NA}}{F_{label}-F_{NA}}\right)\times 100$$

### NanoSIMS

A subset of *P. carrageenovora* samples were analyzed with secondary ion mass spectrometry (NanoSIMS) at Arizona State University to determine single-cell ^13^C values. For preparation of the experimental samples, a total of 900 μL of PFA-fixed cultures (300 μL from each replicate) were pelleted and washed as outlined above for harvested cultures. Cell pellets were resuspended in 500 μL of a 50/50 EtOH/1× PBS solution. Approximately 15–30 μL of resuspended cells were pipetted onto 7-mm square silicon wafers (Active Business Company GmbH), allowed to dry in a culture hood, and then briefly washed in 1× sea salt buffer (SSB) and MilliQ, then dipped in ethanol for dehydration. For comparison to experimental results, an unlabeled *P. carrageenovora* culture was harvested from the late exponential phase, fixed with a final concentration of 4% PFA for an hour, and washed and pelleted following the steps for harvested cultures outlined above.

Masses 24 (^12^C_2_) and 25 (^13^C^12^C) were collected with a cesium ion beam. A mass resolving power (MRP) of > 7,000 was confirmed for ^13^C^12^C. ^14^N^12^C and secondary electron images were also collected to confirm cell area. A 30-μm square area was presputtered with a current of 40 pA for two cycles. Data were acquired with a current of 2 to 4 pA and an area of 25 μm (256 × 256 px; 5,000 cts/px) for at least six frames. Cell ROIs were selected using Look@NanoSIMS (83) with deadtime correction and “new” method alignment. Cell ROIs were quality controlled by viewing secondary electron images (to confirm no overlapping cell ROIs were selected), size (>30 pixels or ~0.6 µm), and Poisson percentage standard error (<5% for all cells). For reference, the minimum cell size was 30 pixels for natural abundance cells. The natural abundance of ^13^C is 1.11%; therefore, the natural abundance of ^13^C^12^C is 2.22% or 0.0222. The average R13 value for PFA-fixed, unlabeled PC cells was 0.0221, with a standard deviation of 0.0003 (*n* = 218), a maximum of 0.0225, and a minimum of 0.0207. The R13 for natural abundance PC cells measured on the EA was 0.01095, or 0.0219 for comparison to NanoSIMS data. The EA natural abundance cells (unfixed) are within one standard deviation of the fixed natural abundance cells measured via NanoSIMS.

Percent enrichment of ^13^C in single cells was determined using Eq. 4. Single-cell ^13^C-DIC incorporation rates were calculated by multiplying the amount of biomass derived from ^13^C-DIC (Eq. 2) in the exponential phase samples by their respective growth rates (Table S3; Table S6). The moles of organic carbon (n_c_) in single cells were approximated using formulas from (84). The volume of ROIs was calculated assuming cells were cylindrical.

### Data processing and statistical analysis

Spearman correlation matrices and associated *P*-values were generated for *P. carrageenovora* and *P. putida* experimental data using the SciPy package and visualized using the seaborn package (85) in Python 3.12. Growth rates (Table S3) were determined using Dashing Growth Curves (86). Figures from this publication were generated using the Matplotlib package in Python 3.12 (87).

## Data Availability

All data presented in this study are included in the article or the supplemental material.

## SUPPLEMENTAL MATERIAL

Supplemental material aem.00628-26-s0001.docx Supplemental text; Fig. S1 to S14.

Supplemental tables aem.00628-26-s0002.xlsx Tables S1 to S7.

ASM does not own the copyrights to Supplemental Material that may be linked to, or accessed through, an article. The authors have granted ASM a non-exclusive, world-wide license to publish the Supplemental Material files. Please contact the corresponding author directly for reuse.
