# Supplementary material for: Heterotrophic inorganic carbon fixation varies with temperature and resource availability in marine and soil bacterial isolates
Source: Appl Environ Microbiol. 2026 Sep 28;92(10):e00628-26. doi: 10.1128/aem.00628-26 (PMC13689363; doi:10.1128/aem.00628-26)
Supplement: Supplementary file 1 [file aem.00628-26-s0001.docx]

**Supplemental materials for**

**Heterotrophic inorganic carbon fixation varies with temperature and resource availability in marine and soil bacterial isolates**

**Figure S1. Left:** SYBR-stained images of *P. putida* cells cultivated in the high organic carbon media (A1) and low organic carbon media (A2) at the optimal (30℃) temperature from time point one (top) to time point two (bottom). **Right:** SYBR-stained images of *P. carrageenovora* cells cultivated in the high organic carbon media (B1) and low organic carbon media (B2) at the optimal (24℃) temperature from time point one (top) to time point two (middle) to time point three (bottom). Average cell size (S_avg_) and average cell circularity (C_avg_) is shown in the top left corner of each image. Statistics of these measurements can be found in Supplementary Data Table 4.

**Cell staining methodology**

To stain cell samples for size measuring, 5-30 μL aliquots of PFA-fixed culture were resuspended in 1mL of 1x sea salt buffer (SSB) to maintain cellular osmotic pressure and then filtered onto 0.22 μm polycarbonate membrane filters. Approximately 0.5mL of SYBR Safe stain was added on top of the filters and then removed via filtration after staining in darkness for at least 15 minutes. Filters were washed with 1x SSB to remove excess SYBR stain. The stained filters were then transferred onto microscope slides for UV fluorescence microscopy using a Leica Epifluorescence Microscope with a 100x objective. For each sample, at least 20 132x132x132 μm fields of view were digitally captured, starting in the bottom left corner of a filter and moving in a stepwise pattern. A field of view was taken at each step until at least 20 images were collected. To measure the cell sizes in each image, DAIME software (Digital image Analysis In Microbial Ecology; (1)) was used. The images were automatically segregated using edge detection to trace cells. These traces were then manually checked for accuracy and redrawn if, for example, many clumped cells were miscounted as one large cell. The “measure objects” function of DAIME was used to specifically measure the total cell area, minimum diameter, and maximum diameter of 100 cells from each sample. For analyzing cell measurement data, a Shapiro-Wilk test (2) was employed to test for normality in the cell area data for all samples, and found that the data were non-parametric. To test for significance, a Kruskal-Wallis test (3) was conducted on the differences between mean cell areas in all *P. putida* and *P. carrageenovora* samples using an alpha-value of 0.01.

**Changes in cell morphology in *P. putida* cultures**

Cultures varied in average cell size and shape depending on the concentration of organic carbon, temperature, and growth phase, with unique trends observed between *P. carrageenovora* and *P. putida* (Supplementary Figure 1, Supplemental Data Table 4). The average cell size for *P. putida* ranged from 0.65 μm^2^ (low organic carbon, T2, 30^o^C) to 4.72 μm^2^ (high OC, T1, 15^o^C). *P. putida* cells from the high OC condition were larger on average (3.25 μm^2^ at 30^o^C; 4.42 μm^2^ at 15^o^C) than those from the low OC condition (1.22 μm^2^ at 30^o^C; 2.32 μm^2^ at 15^o^C) throughout the growth curve. Between *P. putida* cultures grown in the same concentration of organic carbon substrate, those cultivated at 15^o^C had a larger average cell area than their counterparts at 30^o^C throughout the growth curve. All cultures of *P. putida* became more circular between exponential and stationary phases, with cultures grown in lower concentrations of organic substrate generally expressing higher measures of circularity than those grown in high concentrations of organic substrate.

*P. putida* is known to conditionally form filaments, where cell growth continues laterally but no division occurs, which can be seen as an advantage for surviving in a soil matrix (4). Elongation of *P. putida* cells can be seen in all growth conditions, although this is less present in cultures from the low OC condition, especially at 30℃ in the stationary phase (Supplementary Figure 1). *P. putida* cells from a different strain have been shown to shift into a more coccoidal cell shape and decrease in size during carbon starvation, which are both changes attributed to reductive cell division (5). Within the high OC and low OC conditions, *P. putida* cells were more elongated when cultivated at the lower temperature (15℃) than 30℃, which could be explained by a stress response to the cold temperature. Filament formation in response to cold temperatures has been observed in other heterotrophic bacteria (6,7).

**Changes in cell morphology in *P. carrageenovora* cultures**

The average cell size for *P. carrageenovora* ranged from 0.57 μm^2^ (high OC, T2, 14^o^C) to 1.22 μm^2^ (high OC, T3, 24^o^C). Average *P. carrageenovora* cell sizes did not vary to the same magnitude between growth conditions. *P. carrageenovora* cells decreased in average cell size from mid-exponential (T1) to late-exponential/early-stationary (T2) phase in all growth conditions; however, cells from the high organic carbon condition increased in size from T2 to stationary phase (T3), whereas little change was seen during this time for cells cultivated in the low organic carbon condition. *P. carrageenovora* cells at T3 were slightly larger when cultivated at 24^o^C (0.93, 1.22 μm^2^) compared to 14^o^C (0.68, 0.83 μm^2^) when grown with low and high concentrations of organic carbon, respectively. *P. carrageenovora* cells became less circular in stationary phase when grown in high concentrations of organic substrate, and only became more circular when grown in lower concentrations of organic substrate.

While there were similar trends identified in the morphological changes of *P. carrageenovora* cells, the magnitude of cell size and shape changes were minute. *P. carrageenovora* cells from all growth conditions decrease in average size from T1 to T2, but those grown in the low OC condition continue to decrease in size into stationary phase (T3) whereas cells from the high OC condition increase in size. The increase in average cell size in this condition from T2 to T3 is seen in parallel with a drastic increase in C/N ratios and no change to the δ^15^N values of the biomass. An uptake of excess organic carbon into the cell for storage, a process linked to nitrogen limitation, could explain the increase in average cell size in the high OC condition. In contrast to trends observed with *P. putida*, stationary phase cells from the warmer growth temperature (24℃) were larger than those grown at 14℃ in the same OC condition for *P. carrageenovora*. Overall, carbon limitation leads to smaller, more spherical cells in stationary phase for *P. carrageenovora* and *P. putida*, whereas nitrogen limitation leads to larger and longer *P. carrageenovora* cells in high OC condition. However temperature did not have a consistent effect on the cell size between the two bacteriums, which can be explained by different morphological capabilities.

**Figure S2. Top:** Weight percent carbon of *P. putida* (left) and *P. carrageenovora* (right) biomass from batch experiments. **Bottom:** Weight percent nitrogen of *P. putida* (left) and *P. carrageenovora* (right) biomass from batch experiments. Values are calculated from the division of measured micrograms of carbon and nitrogen from EA-IRMS data by the amount of sample weighed out for the analysis. Error bars represent the analytical precision from technical replicates.

**Figure S3.** Optical density measurements of two 50 mL *P. putida* cultures that were grown with minimal M9 media with NH_4_SO_4_ as the nitrogen source and glucose as the sole carbon source. One of the cultures was spiked with filter-sterilized tryptone at 570 minutes. Growth in the tryptone-spiked *P. putida* culture is detected shortly after while no growth is observed in glucose-only *P. putida* culture.

**Figure S4.** Single-cell ^13^C-DIC incorporation rates for *P. carrageenovora* exponential samples cultivated in the high OC condition (red) and low OC condition (yellow) with unlabeled cells (grey) for comparison. Values overlap with DIC incorporation rates measured in crustal fluid incubations (0.042 to 3 fmol C per cell per day) (1).

**Sensitivity of ^13^C-DIC dilution due to organic carbon respiration**

Respiration from cells may dilute the ^13^C-enriched DIC as the experiment proceeds by introducing more ^12^C-DIC from the unenriched organic substrate(s). Therefore, the magnitude of HICF determined via ^13^C-enrichment may be underestimated and will depend on how much ^12^C-DIC is produced by respiration during the experiment – and is likely different in each growth condition. We estimate the amount of respiration in each condition by accounting for biomass generation and applying a range of carbon use efficiency (CUE) values (8). We correlate optical density measurements with the moles of carbon in the freeze-dried pellets using the equation:

$k = \frac{n_{c}}{OD \times V} ,$	(1)

where $n_{c}$ is the moles of carbon in the pellet (calculated from elemental analysis data), OD is the optical density of the culture at the time sampled and V is the volume of the culture. This conversation factor allows us to approximate the amount of biomass generated during each experiment cased on the OD growth curves. The new biomass generated during a time window can be determined by subtracting out previously existing biomass using the equation,

${\Delta n}_{c}=k \times\left( {OD}_{Tf}- {OD}_{Ti} \right)\times V ,$	(2)

where ${OD}_{Tf}$ is the optical density at the time point of interest and ${OD}_{Ti}$ is the optical density of the previous time point. For example, to estimate the amount of biomass generated between inoculation and the first sampling time point, ${OD}_{Tf}$ is the optical density at T1 and ${OD}_{Ti}$ is the optical density at T0. Then, we estimate the moles of respired carbon ($n_{resp}$) based on the CUE using Eq. 3:

$n_{resp}={\Delta n}_{bio}\left( \frac{1}{CUE}-1 \right)$*.*	(3)

Because the CUE is unknown for these strains under the experimental conditions, we apply the range of CUEs (0.2 to 0.8) reported for other heterotrophic bacteria in the literature (8–10): CUE = 0.8 represents a higher , CUE = 0.5 represents an intermediate yield, and CUE = 0.2 represents a lower yield. We included a large range of CUEs to analyze the potential implications of CUE on DIC isotope dilution in our study.

The dilution of the DIC isotope pool ($F_{DIC\_Tf}$) at a given time point can be calculated by using Equation 4:

$F_{DIC\_Tf}= \frac{n_{DIC}F_{DIC\_Ti}+n_{resp}F_{org}}{n_{DIC}+n_{resp}}$ ,	(4)

where $n_{DIC}$ is the initial moles of ^13^C-DIC present in the media, $n_{resp}$ is the moles of respired carbon, $F_{org}$ is the fractional abundance of respired carbon (calculated from the natural abundance preculture sample), and $F_{DIC\_Ti}$ is the fractional abundance of the DIC at the start of the time window you are quantifying. We then determine a correction factor ($d$) as follows:

$d= \frac{F_{DIC\_Ti}-F_{org}}{F_{DIC\_Tf}-F_{org}}$	(5)

Where $F_{DIC\_Ti}$ and $F_{DIC\_Tf}$ are the fractional abundances of DIC at the start and the end of the time window you are quantifying and $F_{org}$ is the fractional abundance of respired carbon.

**Worked example: *P. carrageenovora* at cool temperature (14°C)**

Here we apply this process to both OC treatments for *PC* cultured in the cold temperature (14°C). First, we generate a growth model of optical density measurements using the Gompertz fit given the model’s allowance for asymmetric growth.

**Figure S5.** Averaged optical density measurements for *P. carrageenovora* at 14°C in high OC media (left) and low OC media (right). The optical density data is fitted with the Gompertz model.

Then, we apply Eq. 1 & Eq. 2, to estimate the biomass generated throughout the growth curve using the OD model fit and elemental analysis data from the three samples harvested throughout the growth curve (sample time points are outlined in black).

**Figure S6.** Estimated biomass produced throughout the *P. carrageenovora* experiments at 14°C in high OC media (left) and low OC media (right). The estimates are anchored by pellet weight and Wt%C measurements.

Next, we estimate the amount of carbon respired throughout the experiment over a range of CUE values (Eq. 3).

**Figure S7.** Estimated amount of carbon respired for *P. carrageenovora* 14°C in high OC (left) and low OC (right) media throughout the growth curve. The model predicts more C from respiration in high OC conditions.

We estimate the dilution of the DIC isotope pool ($F_{DIC\_Tf}$) during each experiment as a function of CUE (Eq. 4).

**Figure S8.** Approximated decrease of ^13^C in the DIC pool throughout the *P. carrageenovora* 14°C experiments. The magnitude of ^13^C dilution is sensitive to the CUE value.

Finally, from these curves, we calculate the dilution factor that is used to correct HICF values. This correction assumes cells experienced the averaged $F_{DIC}$ pool over the time interval.

**Figure S9.** Calculated correction factor (d) from Eq. 5 for *P. carrageenovora* 14°C experiments in high OC (left) and low OC (right) media. Larger correction factors are predicted for the high OC conditions.

**Figure S10.** Percentages of biomass derived from ^13^C-NaHOC_3_ in *P. carrageenovora* experiments at the cool T (14°C; left) and optimal T (24°C; right). Solid lines and the associated markers represent the reported values derived from EA-IRMS data. Dashed lines and the associated markers represent estimated values from the calculations above assuming a CUE value of 0.5 (intermediate efficiency) in all conditions. The shaded areas are the estimated HICF values from calculations using a range of CUE values from 0.2 to 0.8. These calculations do not take equilibria of DIC (aq) in the media and CO_2_ (g) in the atmosphere into account.

Applying the full range of correction factors to HICF values reported for *P. carrageenovora* in this study results in higher HICF values up to 3.87x for high OC conditions and 2.16x for low OC conditions depending on the CUE (Fig. S10). Dilution of ^13^C in the DIC pool from respiration could lead to an overlap of HICF activity across temperatures and in high vs. low OC conditions for *P. carrageenovora* when HICF values are closer (e.g., exponential phase in the 14°C experiment). Elsewhere, our estimates reveal that dilution does not explain the different levels of HICF detected in the high vs. low OC conditions for *P. carrageenovora*.

**Worked example: *P. putida* at cool temperature (15°C)**

Here we apply the previously described workflow for *P. carrageenovora* to *P. putida* experiments. Growth curves fitted with the Gompertz model and the resulting estimated biomass generation for *P. putida*, cool T experiments are shown in Fig. S11.

**Figure S11.** Top: Optical density measurements for *P. putida* at 15°C in high OC media (left) and low OC media (right) fit with the Gompertz’s model. Bottom: Estimated biomass produced throughout the *P. putida* experiments at high OC (left) and low OC (right) conditions.

Next, we estimate the amount of carbon respired throughout the growth curves over a range of CUE values (Eq. 3) and then estimate the dilution of the DIC isotope pool ($F_{DIC\_Tf}$) (Eq. 4).

**Figure S12.** **Top:** Amount of respirated carbon estimated for *P. putida* at 15°C in high OC media (left) and low OC media (right). **Bottom:** Approximated decrease of ^13^C in the DIC pool throughout the *P. putida* experiments at high OC (left) and low OC (right) conditions. The initial concentration of ^13^C-DIC is lower and therefore the$F_{DIC}$ is more sensitive to potential dilution from respiration.

Lastly, we calculate correction factors for HICF values.

**Figure S13.** Calculated correction factor (d) from Eq. 5 for *P. putida* 15°C experiments in high OC (left) and low OC (right) media. Larger correction factors are predicted for the high OC conditions.

**Figure S14.** Percentages of biomass derived from ^13^C-NaHOC_3_ in *P. putida* experiments at the cool T (15°C; left) and optimal T (30°C; right). Solid lines and the associated markers represent the reported values derived from EA-IRMS data. Dashed lines and the associated markers represent estimated values from the calculations above assuming a CUE value of 0.5 (intermediate efficiency) in all conditions. The shaded areas are the estimated HICF values from calculations using a range of CUE values from 0.2 to 0.8. These calculations do not take equilibria of DIC (aq) in the media and CO_2_ (g) in the atmosphere into account.

Applying the full range of correction factors to HICF values reported for *P. putida* in this study results in higher HICF values up to 175x and 18x, respectively, for high and low CO conditions (Fig. S14). The ^12^C-CO_2_ generated via respiration is much higher than the starting ^13^C-DIC pool, which results in very large correction factors for *P. putida*. Indeed, respiration drives the DIC pool almost back to natural abundance ^13^C/^12^C ratios (Fig. S13). This is especially prominent in the high OC treatment, where respiration is highest. This causes the denominator in Eq. 5 to get really small, leading to a large correction value. It is important to note that *P. putida* cultures in the high OC conditions were filamentous (Fig. S1) which affects optical density measurements and could lead to erroneous respiration approximations. Furthermore, equilibrium of the DIC (aq) in the media to the atmosphere was not considered in these calculations, which likely results in overestimated ^13^C dilution in the DIC pool given ^12^C is more volatile.

**References**

1.	Daims H, Lücker S, Wagner M. daime, a novel image analysis program for microbial ecology and biofilm research. Environ Microbiol. 2006;8(2):200–13. doi:10.1111/j.1462-2920.2005.00880.x

2.	Shapiro SS, Wilk MB. An Analysis of Variance Test for Normality (Complete Samples). Biometrika. 1965;52(3/4):591–611. doi:10.2307/2333709

3.	Kruskal WH. A Nonparametric test for the Several Sample Problem. Ann Math Stat. 1952;23(4):525–40.

4.	Karasz DC, Weaver AI, Buckley DH, Wilhelm RC. Conditional filamentation as an adaptive trait of bacteria and its ecological significance in soils. Environ Microbiol. 2022;24(1):1–17. doi:10.1111/1462-2920.15871

5.	Givskov M, Eberl L, Møller S, Poulsen LK, Molin S. Responses to nutrient starvation in Pseudomonas putida KT2442: analysis of general cross-protection, cell shape, and macromolecular content. J Bacteriol. 1994 Jan;176(1):7–14. doi:10.1128/jb.176.1.7-14.1994

6.	Shaw MK. Formation of Filaments and Synthesis of Macromolecules at Temperatures Below the Minimum for Growth of *Escherichia coli*. J Bacteriol. 1968 Jan;95(1):221–30. doi:10.1128/jb.95.1.221-230.1968

7.	Mattick KL, Phillips LE, Jørgensen F, Lappin-Scott HM, Humphrey TJ. Filament Formation by Salmonella spp. Inoculated into Liquid Food Matrices at Refrigeration Temperatures, and Growth Patterns When Warmed. J Food Prot. 2003 Feb;66(2):215–9. doi:10.4315/0362-028X-66.2.215

8.	Sinsabaugh RL, Manzoni S, Moorhead DL, Richter A. Carbon use efficiency of microbial communities: stoichiometry, methodology and modelling. Ecol Lett. 2013;16(7):930–9. doi:10.1111/ele.12113

9.	Geyer KM, Kyker-Snowman E, Grandy AS, Frey SD. Microbial carbon use efficiency: accounting for population, community, and ecosystem-scale controls over the fate of metabolized organic matter. Biogeochemistry. 2016 Feb 1;127(2):173–88. doi:10.1007/s10533-016-0191-y

10.	Saifuddin M, Bhatnagar JM, Segrè D, Finzi AC. Microbial carbon use efficiency predicted from genome-scale metabolic models. Nat Commun. 2019 Aug 8;10(1):3568. doi:10.1038/s41467-019-11488-z
